# COVID-19 vaccine knowledge, attitudes, and experiences of health care workers in Perth, Western Australia: A qualitative study

**DOI:** 10.1371/journal.pone.0279557

**Published:** 2022-12-30

**Authors:** Samantha J. Carlson, Sian Tomkinson, Christopher C. Blyth, Katie Attwell

**Affiliations:** 1 Wesfarmers Centre of Vaccines and Infectious Diseases, Telethon Kids Institute, Perth, Western Australia, Australia; 2 School of Social Sciences, The University of Western Australia, Perth, Western Australia; 3 Department of Infectious Diseases, Perth Children’s Hospital, Perth, Western Australia, Australia; 4 Department of Microbiology, PathWest Laboratory Medicine, Perth, Western Australia, Australia; 5 School of Medicine, University of Western Australia, Perth, Western Australia, Australia; University of South Australia, AUSTRALIA

## Abstract

**Introduction:**

Health care workers (HCWs) faced an increased risk of Coronavirus Disease 2019 (COVID-19). Australia’s COVID-19 vaccine rollout commenced in February 2021 to priority groups, including HCWs. Given their increased risk, as well as influence on patients’ vaccine uptake, it was important that HCWs had a positive COVID-19 vaccination experience, as well as trusting the vaccine safety and efficacy data.

**Methods:**

Semi-structured interviews were undertaken with 19 public- and privately-practicing HCWs in Western Australia between February-July 2021. Data were deductively analysed using NVivo 12 and guided by the Capability-Opportunity-Motivation-Behaviour model.

**Results:**

15/19 participants had received at least one COVID-19 vaccine. Participants were highly motivated, mostly to protect themselves and to get back to “normal”, but also to protect patients. Many had a heightened awareness of COVID-19 severity due hearing from colleagues working in settings more impacted than Western Australia. Participants trusted the COVID-19 vaccine development and approval process; their histories of having to accept vaccines for work helped them to see COVID-19 vaccination as no different. Many recalled initially being unsure of how and when they’d be able to access the vaccine. Once they had this knowledge, half had difficulties with the booking process, and some were unable to access a clinic at a convenient location or time. Participants learnt about COVID-19 vaccination through government resources, health organisations, and their workplace, but few had seen any government campaigns for the wider public. Finally, most had discussed COVID-19 vaccination with their social network.

**Conclusion:**

HCWs in Western Australia demonstrated good knowledge about COVID-19 vaccination, with many reasons to vaccinate themselves and support the vaccination of others. Addressing the barriers identified in this study will be important for planning to vaccinate health workforces during future pandemics.

## Introduction

In Australia, vaccination against Coronavirus Disease 2019 (COVID-19) has been free [1]. For the rollout of the vaccine to the population, among a large list of COVID-19 vaccine responsibilities, the Australian Government was responsible for purchasing, identification of and allocation to vaccine priority groups, and developing and delivering national communications campaigns [1]. Other responsibilities (outlined below) lay with the state governments in Australia’s federal system.

When Australia commenced its phased COVID-19 vaccination program in February 2021, care workers (HCWs) were among the first to be offered the vaccines [2]. Globally, frontline HCWs faced increased risks of COVID-19 infection compared with the general community [3] and were deemed priority groups by World Health Organization (WHO) in order to “reduce deaths and disease burden from the COVID-19 pandemic”, and to “protect the continuing functional of essential services” [4]. Initially, Australia’s frontline HCWs were offered either the Pfizer-BioNTech BNT162B2 vaccine (hereon referred to as the “Pfizer” vaccine) or Oxford–AstraZeneca AZD1222 (hereon referred to as the “AstraZeneca” vaccine) as part of “Phase 1a” of the vaccine rollout [5]. As more COVID-19 vaccines became available, “Phase 1b” commenced, in which all other HCWs became eligible for vaccination [6]. Soon into the rollout, the Pfizer vaccine became the “preferred” vaccine for people aged <60 years in non-outbreak settings due to an increased risk of thrombosis with thrombocytopenia syndrome following AstraZeneca vaccination younger cohorts [7]. Eligibility for vaccination was subsequently determined in a staggered approach, where bookings opened up to people in certain age groups based on vaccine supply.

Within Western Australia (WA), Australia’s largest state by land size, and with a population of approximately 2.6 million people [8], residents were able to access a COVID-19 vaccine through General Practitioner (GP)-led Commonwealth Vaccination Clinics, GP clinics, Aboriginal Controlled Community Health Services, community pharmacies, and mass vaccination clinics run by the West Australian Government [9, 10]. At the time of this study, the main state-run mass vaccination clinic available for HCWs to receive a vaccine was located within Perth Children’s Hospital [11], approximately 7km south-west of the Perth Central Business District, but there were other clinics available to healthcare workers and other priority groups. Each vaccination service employed different booking processes, with some walk-ins allowed at the various clinics at the end of each day. At the time of this study, it was also not yet mandatory for HCWs in WA to receive a COVD-19 vaccine, although this policy was introduced for many HCWs on the 1^st^ October 2021 [12].

WA’s COVID-19 vaccination system has been multi-faceted, and it is important to understand the real-world experience of using the system. It was particularly important for HCWs have a positive COVID-19 vaccination experience, as well as trusting the data on vaccine safety and efficacy, so that they could confidently recommend the vaccine to their patients, as well as those in their social networks. A recommendation from a HCW is one of the strongest predictors of vaccine uptake [13].

Globally, the strongest predictor of the acceptance of vaccines in general by HCWs has been the desire to protect themselves [14]. Other strong predictors include the desire to protect friends and family [14]. Pre-pandemic literature has shown that these are also the main facilitators of influenza vaccination of HCWs, along with encouragement and championing by management [15]. A rapid systematic review of the global literature published on HCWs attitudes and acceptance of COVID-19 vaccination identified that gender, age, occupation, race, presence/absence of a chronic health condition, socioeconomic status, level of education, political identification, a history of influenza vaccination, and perceived risk of COVID-19 have all been factors bearing on whether a HCW accepts a COVID-19 vaccine [16]. The most common reasons for COVID-19 vaccine hesitancy amongst HCWs are related to COVID-19 vaccine safety and efficacy concerns [16]. Surveys of Australia’s general population have also identified that gender [17–19], socioeconomic status [17], age [17, 18], level of education [17], private health insurance [18], and the presence/absence of a chronic health condition [18] influence COVID-19 vaccine uptake, but this data is not segregated occupationally. Moreover, most data globally and within Australia is quantitative, meaning that depth of experience and reasons for attitudes cannot easily be discerned.

With little known about the facilitators and barriers to COVID-19 vaccination of HCWs in Australia when we commenced this study, or their experiences of receiving the vaccine, we aimed to study these factors in Perth, WA, during the first six months of the COVID-19 vaccine rollout. WA is of particular interest as a study site due to highly successful elimination strategies that successfully prevented the pandemic from arriving in the state at until early 2022. Short, sharp lockdowns initiated following instances of community transmission, and hard state and international borders in place for almost two years to prevent contact with outbreak locations, the population was largely able to enjoy ‘normal’ life inside the very large state ‘bubble’. As at the 1^st^ December 2021, WA had only recorded 1,111 cases since the beginning of the pandemic in early 2020 [20]. This posed a unique challenge for vaccine acceptance within the state.

## Methods

This study was undertaken as part of the larger *Coronavax*: *Preparing Community and Government* project. Detailed methods have previously been published [21]. In brief, recruitment commenced in late February 2021 through media promotion, word-of-mouth and snowballing. Those interested in participating signed up via a REDCap [21, 22]survey hosted by Telethon Kids Institute, which collected demographic data as well as contact details. If prospective participants identified as working in “health care and social assistance,” and lived within the Perth metropolitan region, they were contacted by telephone or email up to three times each to organise for a face-to-face (at lead author’s workplace), telephone, or videoconference interview. Participants provided written consent and pseudonyms have been used. Ethics approval was granted by the Child and Adolescent Health Services Human Research Ethics Committee (RGS0000004457). One-on-one interviews were undertaken by the lead author (an experienced qualitative researcher with the following credentials: BSc, MPH, PhD), who had no relationship with the participants prior to study commencement. She followed a semi-structured interview guide iteratively developed by the broader Coronavax team, which included generic questions for all Coronavax participants, as well as specific questions for HCWs. Interviews were audio recorded and transcribed verbatim. Recruitment ceased once data saturation occurred. Deductive coding following the Capability-Opportunity-Motivation-Behaviour (COM-B) model [23] was undertaken in NVivo 12. This involved assessing HCWs’:

- Capability: including knowledge and skills to get vaccinated
- Physical Opportunity: the ability to access the vaccine and physical information about vaccination
- Social Opportunity: conversations had and social norms
- Motivation: vaccine attitudes, intentions, emotions, and habitual processes

The lead author undertook the coding and incorporated feedback from all co-authors on the coding framework and subsequent themes. Participants did not provide feedback on the findings.

## Results

Of the 28 HCWs contacted for interview, 19 (68%) were interviewed. Of the remaining nine, four were too busy to be interviewed, four were lost to follow-up, and one was no longer interested. Interviews were approximately 60-minutes in length and were conducted between 3^rd^ March– 5^th^ July 2021. The median participant age was 50 years (range: 27–64 years), many were nurses or midwives (n = 9/19), many worked in public hospitals (n = 9/19) and nearly all (n = 16/19) had direct contact with patients (Table 1). Fifteen (79%) had received at least one COVID-19 vaccine dose at the time of the interview, and the remaining four (21%) intended to be vaccinated. Fifteen (79%) were female, 13 (68%) were born in Australia, all spoke English at home, and only one participant did not have a university degree (Table 1).

**Table 1:** Demographic information of 19 healthcare workers based in Western Australia.

Characteristic	Number (%)
*Received at least one COVID-19 vaccine dose*	
Yes	15 (79)
No	4 (21)
*Occupation*	
Allied health^+^	3 (16)
Doctor	3 (16)
Management (with clinical background)	3 (16)
Nurse or midwife	9 (47)
Paramedic	1 (5)
*Has direct contact with patients*	16 (84)
*Health care setting*	
Ambulance services	1 (5)
Community health	6 (32)
Private clinic	2 (11)
Private hospital	1 (5)
Public hospital	9 (47)
*Has comorbidities*	2 (11)
*Gender*	
Female	15 (79)
Male	3 (16)
Non-binary	1 (5)
*Born in Australia*	13 (68)
*Language spoken at home*	
English	19 (100)
Other	0 (0)
*Highest level of education*	
TAFE/apprenticeship or equivalent	1 (5)
Undergraduate university degree	7 (37)
Postgraduate university degree	11 (58)

^+^Social worker; therapy assistant; complex care coordinator (physiotherapist)

### What facilitated or prevented COVID-19 vaccination among HCWs?

**Fig 1:**
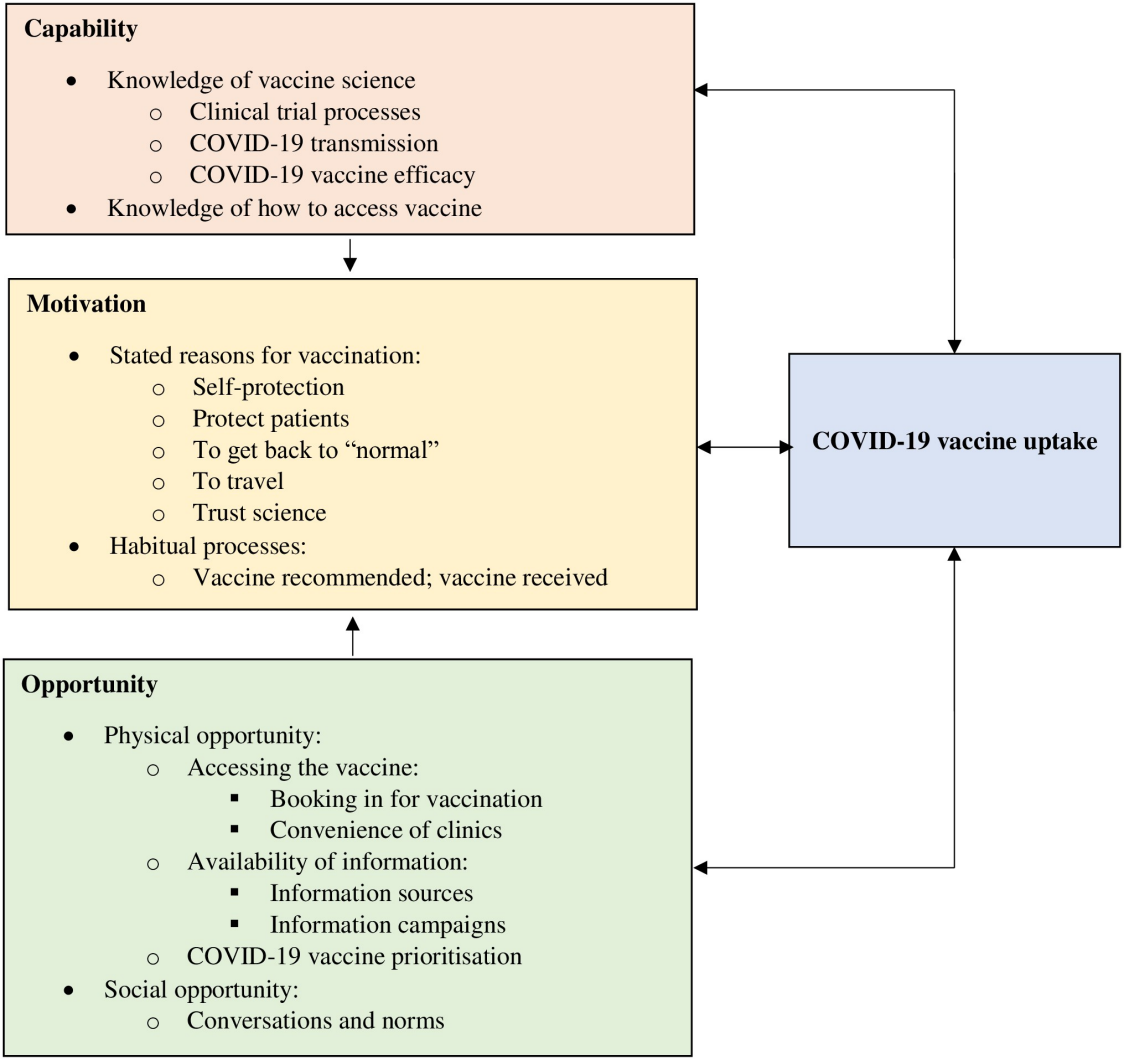
Health care workers capability, opportunity, and motivation to be vaccinated against COVID-19. The arrows depict the way in which each factor influences the other.

Below, we explore HCWs capability, opportunity, and motivation to be vaccinated against COVID-19 within the first six months of the rollout of the vaccine in Australia. Results are also illustrated in Fig 1.

### Motivation

#### Stated reasons for COVID-19 vaccination

HCWs’ most common reason for having already been vaccinated, or intending to be vaccinated, was to protect themselves against COVID-19. This was mostly because of the healthcare setting in which they worked, but also because of concerns about the possibility of a large COVID-19 outbreak occurring in WA. Many participants demonstrated a heightened awareness of this risk, paying attention to large outbreaks occurring overseas such as in the United Kingdom, United States, and Italy. Olivia, a vaccinated anaesthetist who was very positive and confident about COVID-19 vaccination, said:

*We [the anaesthesia department] were watching YouTube videos of doctors in northern Italy where colleagues had died, you know, and I think that idea of that literal threat at work, which for anaesthetists is around what they call “aerosol generating procedures.” So when you intubate somebody, that increases the risk of you actually being exposed to a virus. So I think a lot of that was to do with it being perceived as a real threat, that had already killed people overseas*.

Another motivation for HCWs to vaccinate was to protect their patients. When Bronwyn, a vaccinated nurse, was asked what her main reason for vaccination was, she said:

*Well, one: to protect myself but two: to protect everyone else. Just knowing that…this is the only way forward, that vaccination’s really the only way we’re going to deal with this disease*.

Max, a therapy assistant for autistic children, was highly anxious about passing on COVID-19 to patients, so described their sole motivation for vaccinating as for their patients, rather than themself.

Other reasons for COVID-19 vaccination included to get back to “normal”, as well as to travel within and outside Australia again, given WA had hard border control policies in place at the time

*Being able to travel again is a big one for us, and hopefully travel without having to quarantine. I mean, I can see that being probably a couple of years away, depending on how long it takes, you know, the required amount of people to become vaccinated to do so*

–Sarah, paramedic, unvaccinated

Many were also vaccinated because they trust the process, which includes accepting that science and evidence-based recommendations can change. Bronwyn added:

*…even knowing that the virus will change, I’d still want to be vaccinated, and I think most people that I’m speaking to feel that way as well. Like, well, this is what we’ve got, and this is the best answer we’ve got at the moment, so we’ll go with that, and hopefully science just keeps working on a better vaccine*

–Bronwyn, nurse, vaccinated

#### Habitual processes

*Vaccine recommended; vaccine received*. Most participants had a history of accepting whichever vaccine was recommended:

*If a vaccine is recommended I will get it. I’m so one hundred percent pro-vaccine that I will take anything that’s on offer, really*

–Toby, doctor, vaccinated

Some mentioned travel vaccines, the vaccines received in school, and booster shots. The most common “habitual” vaccine referred to, however, was the influenza vaccine, which was encouraged and offered annually at their workplaces (but not mandated, except in aged care). Only one participant stated outright that she doesn’t receive an annual influenza vaccine:

*Now for me in emergency, I don’t ever get the flu vax ever. I’m a conscientious objector, because I am 57 years old, I still run marathons, I surf, I am fit and healthy and I never, touch wood, get sick. So why would I vaccinate myself with a flu vax that changes every year, when I work in the front line, I deal with the flu people all week and I just don’t seem to get sick, I have a good immune system. When I get older and start getting sick, then probably I will have the flu fax, but at the moment I don’t think I need it. Now whether that’s logical or not doesn’t matter. But COVID is completely different, it’s going to kill you and you’re going to kill other people, because that’s how serious it is*

–Esmerelda, nurse, vaccinated

Potentially due to this “vaccine recommended; vaccine received” behaviour, very few participants had concerns about vaccine brands and were happy to accept whatever vaccine was offered. Matt, an allied health worker vaccinated with AstraZeneca, said he would still accept the vaccine “today, even given the very rare blood clotting.” However, other participants did specifically seek out Pfizer over AstraZeneca. This was either due to AstraZeneca safety concerns and changing recommendations, or because the shorter timeframe between Pfizer doses was more appealing. In some cases, changes to advice and availability motivated participants to vaccinate at a particular moment.

*I always thought I wanted to get Pfizer if at all possible. So I just delayed … people were getting it around me at work and I just didn’t want to, I was a little bit scared of it. And then when I found out that they were…going to make people 50 age and over have the AstraZeneca and also if you’d had a Pfizer appointment booked they were gonna cancel it on you, I was just like: “Absolutely no way am I having that choice taken away from me!” So I took the last minute vacc[ination] appointment that I could at…a Pfizer clinic that was being offered to the Department of Health staff before the 50 plus cut-off*

–Zoe, allied health, vaccinated (aged 51 years)

### Capability

#### Knowledge of vaccine science

There were mixed responses in how knowledgeable participants felt about COVID-19 vaccination. However, many described the COVID-19 vaccine development process and the associated clinical trials, understanding that the vaccines were produced “quickly” not due to rushed science, but due to less bureaucracy.

*The technology was there and had been used already, it wasn’t new… The same processes that every vaccine goes through went through, it’s just that this time money was just thrown at them … so they weren’t having to apply for funds and then go through a trial and then apply for the next lot of funds to do the next process. They just had money to go from one process to the next and it was just streamlined. And also the red tape was cut, so that when they were trying to get approvals and things through, you know, they weren’t held up…*

–Jacinda, nurse, vaccinated

Many also described how vaccinated people can still get a less severe COVID-19 infection and can still transmit the disease. Zoe, a vaccinated allied health worker said:

*I think everyone’s kind of conscious the vacc[ination] is not going to stop it, and then we’re all maybe concerned … they’ll open up the borders again and everything will return to, you know, “normal,” but that it will then be rampant in the community*.

Some also quoted what they believed to be the specific efficacy of certain brands, and others shared their knowledge of COVID-19 vaccine safety, mostly in relation to the risk of blood clots.

#### Knowledge of how to access vaccine

Many participants, especially those interviewed early in the project, were unsure of when they would be able to access the vaccine, mostly due to a lack of government or employer communication about which vaccine priority group they fell into. Some were waiting to be told, such as Steph, an unvaccinated nurse:

*I have no idea of when that’s happening*. *A friend who works in ICU has had hers done*. *But I have no idea*, *I wouldn’t even know if it was next month or in the next four months*, *five months*, *there is no knowledge of when*.

Others, employed in either public health settings or private practice, had to actively seek out information:

*…for a while we weren’t sure if we were eligible or not and then we were like "oh no wait, we are eligible? OK, what are we gonna do?" [Then] we got an email from head office saying… "some of you will be eligible to receive this vaccination, we will let those people know." It was a little bit tricky because they let some of the people know but not all of them and so some of my colleagues were like "oh yeah, so I got my vaccine today" and I’m like "what, I haven’t gotten my letter to say I’m eligible yet." But then when I emailed [Human Resources] they were like "oh no, you are, here’s your letter"*

–Max, allied health, vaccinated

Once participants did find out how to access the vaccine, some then did not know which vaccine brand they received until after they had received it.

### Physical opportunity

#### Accessing the vaccine

*Booking in for vaccination*. While half of the participants described the booking system as “simple” (Florence), “painless” (Emma), or could easily book over the phone (Zoe), the other half discussed the difficulties in navigating the process, describing it as a “nightmare” (Toby). Some received an email inviting them to be vaccinated but the link to register did not work. Others were unable to open the link on certain browsers, and Carole described having to call a support line at WA Health three times in order to log in to the booking website. Bronwyn said *“that a lot of people had difficulty using the booking system*.*”*

*Convenience of clinics*. Some participants were able to be vaccinated at a COVID-19 vaccine clinic coincidentally within their workplace; others received a vaccine at a clinic near their home. Emma, a midwife/nurse vaccinated outside her workplace, said:

*I found the process easy. I’m also fine with technology, have a car, pay for parking, don’t have children, can go on a day off, like I’ve got all of these things that make it really easy and accessible for me*.

However, other participants were waiting for the vaccine to be given within their workplace; they could not be “bothered” driving into the city when they anticipated the vaccine would at some stage be offered at their workplace (Francine). A minority actively sought out clinics that provided the Pfizer vaccine.

Many participants received the vaccine on a day off from work, even if they received it at a clinic within their workplace. Bronwyn, a vaccinated nurse who works in a public routine-vaccination clinic, said:

*They had said at work, “please try and go in your own time, go in your own day off”… but the only appointment that I could get was actually on a clinic day. So my colleague and I were quite lucky, we both got an appointment around the same time and that was at [hospital clinic] for our second one and we just rejigged our clinic and went in in the afternoon and had our second vaccine*.

Esmerelda, a vaccinating nurse working in a public emergency department, shared a similar experience:

*When we got vaccinated, we got an email to say: could we do it on our days off, so that we didn’t interrupt the roster in case we got sick*.

When Bronwyn was asked how she felt about this request, she said:

*On one hand I can understand, they’re really short-staffed and they’re finding it really hard to backfill a lot of our clinics and they’re cancelling some of our infant clinics. But at the same time, I was like it’s really important that we’re immunised and sometimes you’ve just got to take the appointment that you can get. And that’s exactly what we did. I couldn’t get one on my day off, so I did just have to go during work time*.

The majority of participants were satisfied with the service at the clinics. Olivia said that the waiting room was “very upbeat….it just felt really like an opportunity, very positive.” Matt said his mass vaccine clinic “was far and away the best vaccination system I’ve had so far.” However, Max, who was vaccinated during a lockdown, said the waiting room was “pretty sedate.”

Participants were also satisfied with the care from those providing the vaccines, and in the 15-minute time period for which those who have been vaccinated are encouraged to stay around. Jacinda’s vaccinator was “very respectful” of her needle-phobia. Lucy, who was immediately seen upon arrival to the clinic for the first vaccine dose, said of her second dose:

*the girls checked me in, I did wait that time to be seen, and again she did her checks, made sure I didn’t have any of the clotting disorders and then sent me out where another lovely lady gave me an apple juice*.

#### Availability of information

*Information sources*. Participants accessed a wide range of COVID-19 vaccine information sources. Non-media included government websites; academic articles; health organisations such as the National Centre for Immunisation Research and Surveillance or the Australian Medical Association; and information provided through workplace emails, online training, and the intranet. Many participants said that they don’t consume media (television, radio, newspaper), but then went on to acknowledge that they at times actively seek out their news from ABC, the national public broadcaster, either on the television, on the ABC mobile phone app, or via a COVID-19-specific daily podcast produced by the ABC called ‘Coronacast.’ Participants were highly skeptical of mainstream media, including the ABC, despite watching it:

*The media needs to jolly well be gagged…All they’ve done is sensationalise it, and they have been the drivers of the fear. They’ve been in a position where they could really positively impact and they haven’t*

–Jacinda, nurse, vaccinated

Kate was of the belief that the media should be held to higher standards:

*They go on about Facebook not taking down rubbish and fake news, but I think the media platforms, on their online media platforms where people comment, they shouldn’t be publishing fake news as well… The government monitors the media–I think they should be reporting them for breaching media standards. Because they have a [moderator] that determines what comments to get published, so they’re not doing their job right. It’s not free speech when you’re spreading dangerous information in a pandemic*

–Kate, management, vaccinated

Participants could also recall seeing and sharing COVID-19 vaccine information on Twitter, Facebook, Instagram, LinkedIn and Reddit, including COVID-19 vaccine selfies. Participants often saw negative information, especially on Facebook, but also praised official links to learn more about COVID-19:

*you often see these little things come up saying “click here to find out more about COVID”, and that’s a good thing*

–Jason, management, vaccinated

*Information campaigns*. Very few participants could recall seeing any government COVID-19 vaccination information campaigns; either by the WA state government or the Australian government. Carole recalled a television advertisement with “*different health professionals saying la*, *la*, *la*, *go and get your vaccine*”; Jacinda and Florence vaguely recalled the ‘Roll Up for WA’ campaign; and Bronwyn recalled hearing on the radio that vaccines are important and safe. No participants could recall seeing any visual advertisements in the community, such as on public transport.

We asked all participants how governments could improve information campaigns; the majority suggested a focus on vaccine safety, and normalising minor side effects in the days after vaccination. Other suggestions included information on vaccine efficacy, the vaccine development process, on the proportion of Australians who had been vaccinated, on local epidemiology of the virus, and how to actually go about accessing the vaccine. Another angle could involve personal stories about why it’s important to be vaccinated, such as to get back to “normal,” to travel, and to protect your family.

*They need to reassure people that the vaccine is safe, effective and protects them against Coronavirus. That’s the first bit. And the second bit is if we’re going to step back to normal life, you need to get vaccinated. And delaying it is only going to delay our ability to return to free travel and no lockdowns and all that kind of stuff. So the consequences of not getting vaccinated are: we all stay like this for longer, and you risk getting sick from Coronavirus*.

- Toby, doctor, vaccinated

Participants also described the way information should be presented in a campaign. Several referred to needing to “*counteract …sensationalist*” media messages (Jason), and for it to be “*really positive and hopeful*” (Zoe). They suggested a visual breakdown of the scientific information, and for it to be presented by “normal” people from a diverse range of backgrounds, as well as health professionals or scientists. While the majority recommended that politicians not be featured in campaigns, one participant did suggest the extremely popular current WA premier, Mark McGowan, widely lauded for keeping COVID-19 out of the state, could be the face. Jason suggested that politicians with a “standing throughout the whole community” (and then referenced previous Australian Prime Ministers such as John Howard and Paul Keating), and others provided ways in which politicians *could* be the face if absolutely necessary:

*Like, if you look at Mark McGowan’s Facebook page, he has done an incredible…service with the information that he puts out and the followers and the people that, you know, and it could be like some of those real champions of people, not just the “look at me, like I got the COVID shot, look aren’t I great,” which you [saw when] Julia Gillard [past Australian Prime Minister] got hers and ScoMo [past Australian Prime Minister, Scott Morrison]. You want those real like “yeah my arm was sore but that’s OK… I was achy for the first two days but you know what, that’s OK.” And really normalising, we want people to normalise the process, not just “look at me, I got the shot.”*

- Steph, nurse, unvaccinated

### COVID-19 vaccine prioritisation

The majority of participants accepted the prioritisation of certain groups for vaccination. We spoke to people in phase 1A (such as a paramedic, a nurse working in a COVID-19 testing clinics, and nurses and doctors working in hospital EDs), as well as phase 1B (all other HCWs). The majority, however, felt that the communications about exactly who was a frontline worker were not clear. This caused some confusion among themselves and their patients (see *Capability* for more on this). A couple of participants felt “*uncomfortable*” in being told they were a priority, such as Carole who worked in COVID-19 testing clinic:

*I mean obviously we are frontline, we are supposedly testing people with COVID. But because we don’t have any cases, you do feel like a bit of a fraud*.

Olivia, an anaesthetist in a public hospital setting, felt “*very positive*” about being vaccinated sooner than expected as someone in Phase 1B. Sammy, who works in management, had not yet been vaccinated as “*I’m not getting called up*,” and was “*happy*” to keep waiting due to an underlying concern about COVID-19 vaccine safety. Florence, an ED nurse in a “*private hospital that works for the government*” felt that workers in public settings were prioritised over those working in private settings. She also said that doctors were originally able to choose which vaccine brand they were able to receive, but nurses could not.

### Social opportunity

#### Conversations and norms

In our probing of their role as early adopters and social influencers, participants recalled conversations with friends and family members about COVID-19 vaccination. Topics included possible side effects, opinions on the COVID-19 vaccine rollout in Australia and elsewhere, being able to travel again, and people’s opinions on COVID-19 vaccine brands.

Many of these memorable conversations happened at dinner parties, or around the family dining table as a “Sunday night dinner conversation.” Several participants reflected on friends and family members being unsure of or against COVID-19 vaccination, and some went on to recommend vaccination to them. with several participants saying that they gave a recommendation to vaccinate.

There was some reflection on the position of HCWs in a social setting hearing about people’s misguided views. When asked whether she knew how to respond to friends and family questioning the safety of COVID-19 vaccines, Steph said:

*I just say I would be happy to get it. And it’s an awkward– … conversations about vaccines are almost as difficult as conversations about people’s religious beliefs. You know, you don’t want to offend anyone, but at the same time you’re still, like, “Oh, come on.” So it’s hard to influence or change people’s minds sometimes, socially, when they have their own opinion*.

Only one participant recalled a conversation in which *they* were influenced by their social setting, rather than trying to influence others. Zoe, who had some concerns about different brands even before the vaccine program commenced in Australia, said:

*I’ve got a close friend who is a … he’s a virologist, he specialises around virology, like viruses [are] his thing. So I remember talking to him about it and saying, “What would you recommend in terms of vaccines?” And he said Pfizer*.

Very few participants consulted with a general practitioner (GP) for advice on their COVID-19 vaccines. Lucy discussed her concerns about blood clots with her child’s GP following her first dose of the AstraZeneca vaccine, and Max, who had organised to be vaccinated at a GP clinic prior to the pivot to Pfizer for their age group, said:

*I had booked into get my AstraZeneca vaccine but then of course they cancelled it so that was annoying. The practice set up a telehealth appointment for me so that I could talk to her about the vaccine and whether it was OK for me, because she knows me and my medical history, so she can answer those questions*.

Conversations with colleagues focused on vaccine accessibility, personal experiences of receiving the vaccine including needing to take time off after vaccination, as well as safety and efficacy.

*a group of my colleagues were having coffee and we were discussing it… It has been a variety, because some people have had more systemic, you know, nothing serious but have … gone to bed for a few days with sort of flu symptoms, have come off work for maybe a couple of days, and some who just had local ache. Most people have had a little bit of an ache in the arm but nothing, you know. I don’t think I’ve had anyone who’s had absolutely nothing*

- Alma, doctor, vaccinated

Some participants also knew of colleagues who had no intention to be vaccinated (unless it became mandatory), or were being selective about brands. Jason, who was interviewed during a lockdown in Perth, was in the minority in reporting that he and his colleagues do not discuss COVID-19 vaccination:

*it’s not a topic of conversation particularly, except like today with the masks and everything [due to lockdown], conversation revolves around "oh bugger here we go again." But we all know it’s for the good, and we just get on with it… In the scheme of things in what we do in the hospital it’s not big, it’s tiny*.

Despite the difficult conversations, many participants believed that their friends, family and colleagues would get vaccinated when they could.

## Discussion

Overall, our HCW participants in Perth demonstrated good COVID-19 vaccine knowledge and were highly motivated to be vaccinated. They trusted the data on COVID-19 vaccine safety and efficacy, which was not the case for HCWs in all comparable global settings (e.g. the UK’s ethnically diverse health workforce [24] and health workers in Hong Kong [25]). Our participants felt confident that vaccination would protect themselves and others around them, enabling the community to get back to “normal.” HCWs in other countries (e.g. Canada [26] and the UK [24]) and other Australian states [27, 28] echoed similar drivers, particularly normality. A study in the eastern Australian state of Queensland identified that participants’ belief that close family and friends would want them to get vaccinated was significantly associated with uptake [28]. While we did not explore this directly, we saw mutuality flowing in the other direction: our participants felt that those closest to them would get vaccinated, and some were actively encouraging this, as also seen in HCWs in other global settings [24, 26]. We note that “normal” for our participants was a world free from pandemic control measures which were successfully keeping the disease out of the community, whereas HCWs in countries such as the UK embraced vaccination as a way out of the pandemic because other prevention and control measures had failed [29]. This difference likely informed other ways that our findings deviated from global studies–for example, some of our participants felt ‘fraudulent’ about receiving priority vaccines in a non-outbreak settings, whereas HCWs facing imminent threats overseas appreciated being prioritised [29].

Many of our participants reported receiving COVID-19 vaccine information through non-media sources, such as academic articles and information developed by health organisations, often formally shared through their workplaces. Their main media source was the ABC, Australian’s national broadcaster, and most had not seen any Government information campaigns. We conducted many of our interviews at the beginning of the COVID-19 vaccine rollout in Australia, before any official Government information campaigns commenced. For example, the state campaign ‘Roll Up For WA’ was launched two months into the COVID-19 vaccine rollout [30]. The trusted national broadcaster appears to have been an effective conduit to reach those who were not actively accessing information through their workplace. Further, given some of our participants were confused about which priority group they were in, clear guidance on who can be vaccinated, when, and how, is required in ongoing communications and will be of central importance to booster regimes.

As identified in other global studies [16, 29, 31], a history of receiving the influenza vaccine was a strong motivational factor for many our participants. According to internal communications from WA Department of Health, 81% of HCWs received an influenza vaccine in 2020 [32] without a mandate, while the community waited for a COVID-19 vaccine. Governments and healthcare settings can learn from the rollout of COVID-19 vaccines how to improve influenza vaccine uptake for HCWs like Esmerelda, who were only receptive to COVID-19 vaccination. Likewise, success stories from HCW influenza vaccination can be applied to COVID-19 vaccination in this population, including incentives, mandates/incentives, and multi-component interventions [33].

Many of our participants reported difficulties in being able to make a vaccine booking and being able to access a clinic, inside or outside of work hours. While we acknowledge that there are complexities given Commonwealth, State, and private provider responsibilities in the COVID-19 vaccine rollout, once it became mandatory for HCWs in WA to receive a COVID-19 vaccine [12], ensuring HCWs can either be vaccinated during their work hours on-site, or giving HCWs paid time off to get vaccinated, became paramount [34]. Though there are HCW shortages in WA (as referred to by Bronwyn), allowing the remaining minority of HCWs who aren’t vaccinated to take some time during a shift to get vaccinated at a clinic within their workplace would likely not be so burdensome to the health system. Finally, although there are inevitably teething problems, reviewing and simplifying the booking process early and quickly would have enabled HCWs, and others in the WA community, to get vaccinated more easily.

While our study did not capture the voice and experience of those who refused the COVID-19 vaccine, it was quite representative of the COVID-19 vaccine uptake by HCWs in Perth, given that 79% of our participants had received at least one dose by July 2021, compared with 85% of frontline workers in Perth receiving the COVID-19 vaccine as of the end of July 2021 [35]. It may be, like our four unvaccinated participants, that many ‘hold out’ healthcare workers were waiting for easier access to the vaccine, or were waiting until it was mandatory to be vaccinated in their work place–a policy which did eventuate [36].

## Conclusion

Overall, HCWs in Perth, WA, had good knowledge about COVID-19 vaccination, and many reasons to vacciante, including to protect themselves, protect others, and to get back to “normal” (such as being able to travel again). Our interviews were conducted in the first six months of the COVID-19 vaccine rollout: with this, there was some confusion about accessing COVID-19 vaccines, and difficulties in using the booking system. Attending to the types of motivational and access barriers identified in this study is crucial in any healthcare context globally, particularly now that COVID-19 vaccination of HCWs made mandatory in many settings.
